# A self-guided digital mental health intervention for Syrian refugees in Germany and Sweden: effects from two pragmatic randomized controlled trials

**DOI:** 10.1186/s12888-026-08241-4

**Published:** 2026-05-29

**Authors:** Sebastian Burchert, Mhd Salem Alkneme, Ammar Alsaod, Kenneth Carswell, Pim Cuijpers, Anne M. de Graaff, Eva Heim, Jonas Hessling, Tomas Lindegaard, Shervin Shahnavaz, Marit Sijbrandij, Edith van’t Hof, Mark van Ommeren, Christine Knaevelsrud

**Affiliations:** 1 Department of Education and Psychology, Division of Clinical Psychological Intervention, Freie Universität Berlin, Berlin, Germany; 2 German Center for Mental Health (DZPG), Partner Site Berlin-Potsdam, Berlin, Germany; 3 Department of Systemic Psychotherapy, Berlin School of Psychology, Berlin, Germany; 4 Department of Noncommunicable Diseases and Mental Health, World Health Organization, Geneva, Switzerland; 5 Department of Clinical, Neuro and Developmental Psychology, WHO Collaborating Center for Research and Dissemination of Psychological Interventions, Amsterdam Public Health Research Institute, Vrije Universiteit Amsterdam, Amsterdam, The Netherlands; 6 International Institute for Psychotherapy, Babeș-Bolyai University, Cluj-Napoca, Romania; 7 Institute of Psychology, University of Lausanne, Lausanne, Switzerland; 8 Department of Behavioural Sciences and Learning, Linköping University, Linköping, Sweden; 9 Centre for Psychiatry Research, Department of Clinical Neuroscience, Karolinska Institutet, & Stockholm Health Care Services, Region Stockholm, Stockholm, Sweden

**Keywords:** Digital mental health, e-mental health, Self-help, Smartphone-based, Syrian refugees, Syria, Scalable interventions

## Abstract

**Background:**

Syrian refugees across diverse host countries, including high-income European countries, face increased mental health needs. Digital interventions can scale support, but global scalability limits human guidance and contextual adaptations. We evaluated the effectiveness of a potentially scalable digital intervention (Step-by-Step; SbS) with minimal contact-on-demand (COD) in reducing psychological distress and functional impairment among Syrian refugees in Germany and Sweden. These trials were conducted in parallel with SbS studies in Egypt and Lebanon, using the same content to test broader contextual applicability without further adaptations.

**Methods:**

Separate two-arm pragmatic RCTs were conducted in Germany (*N* = 559) and Sweden (*N* = 184) with Syrians screening positive for elevated distress (K10 > 15) and impaired functioning (WHODAS 2.0 > 16). Participants were randomized to SbS (five sessions) + care-as-usual (CAU) or CAU-only. Primary outcomes were psychological distress (HSCL-25) and functioning (WHODAS 2.0) at 3-month follow-up. Secondary outcomes were PTSD symptoms (PCL-5 short) and self-defined problems (PSYCHLOPS). Intention-to-treat (ITT) analyses were run separately by trial. Exploratory per-protocol analyses combined datasets.

**Results:**

ITT analyses showed no statistically significant time × condition effects for any primary or secondary outcome in both trials. Dropout was high (Germany: 86.3%; Sweden: 82.1%). In per-protocol analyses (participants completing ≥ 4 of 5 sessions), the SbS + CAU arm showed significantly lower standardized mean scores at 3 months for psychological distress (HSCL-25; Hedges’ g = 0.31; *p* = .03) and PTSD symptoms (PCL-5 short; Hedges’ g = 0.27; *p* < .05). COD use was low (Germany: 15.1%; Sweden: 8.4%), leaving the intervention effectively unguided for most participants.

**Conclusions:**

While limiting guidance and contextual tailoring can enhance scalability across borders, digital interventions may struggle with engagement, adherence, and contextual relevance. In high-income settings, an unguided approach for refugees may not work, showing that prioritizing scalability could potentially compromise clinical impact in this population. Some level of human guidance may be necessary to balance scalability and effectiveness, and it remains unclear how minimal that guidance can be without compromising outcomes.

**Trial registration:**

German Register for Clinical Studies (Germany: DRKS00022143—registered June 29th, 2020, and Sweden: DRKS00022144—registered July 1st, 2020).

## Background

The conflict in Syria has caused the displacement of a substantial percentage of the Syrian population with up to 5.5 million registered Syrian refugees in host countries throughout the world [1]. While most Syrians fled to neighboring countries like Türkiye or Lebanon, over 1 million Syrians were displaced to Western European countries. At the peak of Syrian refugee movement to Europe in 2015 and 2016, Germany and Sweden became the two largest host countries, with up to 70% of the Syrian refugee population in Europe [2].

Surveys on the prevalence of Posttraumatic Stress Disorder (PTSD) symptoms and symptoms of depression in refugees in Germany are heterogeneous but aggregated data points towards rates of 29.9% for PTSD and 39.8% for depression [3]. Almost identical results were found in Syrian refugees in Sweden with 29.9% for PTSD and 40.2% for depression [4]. These findings align with data from other major countries hosting Syrian refugees, such as Lebanon, Jordan, and Türkiye [5]. Long-term, psychological distress has been shown to negatively affect everyday functioning in refugees [6]. Addressing these mental health needs in host-country healthcare systems is therefore essential in preventing further symptom deterioration, chronicity, as well as commonly reported difficulties in handling postmigration stressors that impact mental health [7].

Host countries differ substantially in how they organize refugee healthcare [8]. This variation shapes the services defined as care-as-usual (CAU), which may also include no care at all because of context-specific or general access barriers. In Germany, the public health system offers general and specialized care for refugees, but in practice, access is restricted by legal and practical barriers. For a period of roughly 1.5 years, newly arrived refugees only have access to treatments for acute illnesses and pain as part of restricted healthcare benefits. During this time, mental health care is provided to some through psychosocial organizations specialized in refugee care, but these have long waiting lists and do not exist in all regions [9]. After general care becomes accessible to refugees, barriers include language, a lack of culturally-trained professionals, and limited financial resources for interpreters [10]. Specialized mental health programs that are culturally adapted and available in the Arabic language remain scarce. Similarly, in Sweden, general and specialized healthcare is available for refugees, but structural limitations hinder effective mental health support. By law, asylum seekers receive a voluntary health assessment and care that cannot be postponed, yet these assessments often fail to detect psychological needs [11], resulting in missed referrals. Furthermore, a shortage of interpreters complicates treatment for Syrian clients [12].

The EU-funded STRENGTHS project aimed to strengthen mental healthcare systems for Syrian refugees by evaluating a range of potentially scalable interventions developed by the World Health Organization (WHO) and partners [13]. These included individual, group, and digital formats, such as the Step-by-Step (SbS) intervention, and were tested in several host countries, including Germany and Sweden [14]. A digital intervention was identified as a potentially scalable solution for Germany, Sweden as well as Egypt [15] to support local healthcare systems and provide location-independent assistance to a large number of individuals who would otherwise receive no support due to structural limitations and barriers.

Digital mental health interventions (DMHIs) can provide mental health support for hard-to-reach populations by providing low access thresholds, flexible use, anonymity, and reduced stigma [16]. While evidence suggests they can work in low-resource settings, research involving Syrian refugees is limited, with effect sizes ranging from non-significant to moderate [17]. Despite a heterogeneous and relatively weak evidence base for interventions specifically designed as smartphone apps for refugees and migrant populations [18, 19], such app-based approaches may offer unique advantages for those who have limited access to infrastructure, for example by enabling use without constant internet connectivity. Although these interventions are often viewed positively, past trials have faced challenges in recruitment and adherence [18, 20, 21]. These obstacles may be mitigated through further cultural and contextual tailoring [22].

The initial version of SbS was developed by WHO to create a low-threshold and potentially scalable DMHI for populations with limited access to care while living under adversity [13, 23]. A revised version of SbS focused on the contextual adaptation for Syrian, Lebanese, and Palestinian populations. It was developed in collaboration between WHO, National Mental Health Programme of the Ministry of Public Health, Lebanon, Freie Universität Berlin, and University of Zurich [22, 24]. The SbS intervention platform was adapted for use on mobile devices and equipped with distinct features to improve usability and accessibility, including full audio support for illiterate users and offline capabilities for limited access to the internet.

There are two distinct guidance models with which SbS has been offered so far. Two RCTs in Lebanon implemented guided SbS offering scheduled 15-minute weekly contacts with trained non-specialist helpers, through phone or in-app messaging. These studies found small to medium-sized effects on symptoms of depression, anxiety, and PTSD as well as impaired functioning, well-being, and burden from personal problems [25, 26]. As part of the STRENGTHS project, a study was conducted with Syrian refugees in Egypt, following the same study design and procedures described for the STRENGTHS Germany and Sweden studies in this paper. In all STRENGTHS trials, scheduled weekly guidance—as done in Lebanon—was replaced with contact-on-demand (COD) to increase the scalability of the intervention. In self-guided SbS with COD, participants can send in-app text or audio messages to a team of trained and supervised e-helpers providing support with specific questions about the intervention, the study, technical issues, or referral to other services. The Egypt study found small effects on psychological distress and impaired functioning, however, compared with studies on the guided version, effects of the self-guided version of SbS were smaller, and intervention drop-out rates were slightly larger [15].

The main objective of our trials was to evaluate the effectiveness of SbS under real-world conditions, allowing participants to use other services in parallel. By keeping exclusion criteria to a minimum, we aimed for a heterogeneous sample. The aim was to compare changes in psychological distress, functional impairment, symptoms of PTSD, and burden from self-defined problems between Syrians with access to SbS and those without. These two studies in Germany and Sweden complement the existing SbS literature by adding the first data on implementing the intervention for Syrian refugees living in high-income host countries, thereby diversifying the evidence base for this digital intervention approach.

## Methods

### Design

Two parallel two-arm randomized controlled trials (RCTs) were conducted, sharing the same study design and procedures. They are reported according to the Consolidated Standards of Reporting Trials (CONSORT) guidelines [27]. Participants were randomly assigned to either the self-guided version of SbS plus CAU or to a CAU-only group. CAU was defined as any treatment participants had access to, including primary care, specialized care, medication, alternative care, or no care. Study group allocation was implemented to happen automatically and fully independent from the study team, using a permuted block randomization algorithm with 1:1 allocation and random block lengths of 2,4, 6, or 8.

### Participants

Arabic-speaking Syrian refugees residing in Germany and Sweden with a basic level of literacy in Arabic and access to the internet (either on a mobile device running Android or iOS, or on a standard web browser) were eligible to participate in the studies. Further inclusion criteria were assessed with screening questionnaires and included elevated levels of psychological distress (Kessler Psychological Distress Scale; K10 > 15) [40] and impaired psychosocial functioning (WHO Disability Assessment Schedule; WHODAS 2.0 > 16) [41]. Criteria for study exclusion were being under 18 years of age and self-reporting serious suicidal thoughts or a plan to end one’s life.

### Procedure

In Germany and Sweden, participants were initially and mainly recruited through social media ads, outreach in social media groups, and by contacting government and non-government stakeholders with direct contact to refugees (e.g., citizens’ offices or language schools). Later, recruitment efforts were shifted towards Syrian social media influencers who were approached as mental health ambassadors in a coordinated campaign consisting of a multi-month series of social media posts that incorporated information on the STRENGTHS project into their regular social media content. On all recruitment channels, links to the project landing page were distributed that provided potential participants with a short introduction to SbS, information about the research, as well as links to the iOS and Android mobile versions and the SbS web version. Once downloaded or accessed via a web browser, SbS automatically guided participants to study information, data protection information, and an informed consent form. Participants then created an account by picking a username and setting a password. This was followed by a set of screening questionnaires. Those not matching the inclusion or the exclusion criteria were excluded from participation in the study and instead received referral information to other services.

### Study arms

Participants in the SbS + CAU arm could use any additional services available to them in their country of residence (or online) and, over 6 weeks, received access to the standard set of 5 SbS intervention sessions plus an introductory session. Participants were encouraged to complete one session per week and to practice the techniques learned between sessions. This setup and dose were consistent with previous SbS studies [15, 25, 26]. The SbS intervention primarily focused on behavioral activation and stress management techniques, adapted for smartphones through interactive exercise interfaces (e.g., creating and storing lists), a mood tracker with an automatic display of mood curves, and an activity planner with a notification feature that reminded participants of scheduled activities. In addition, SbS provided psychoeducation and encouraged participants to strengthen their social support networks. The fifth SbS session served as a review of these techniques and included elements of relapse prevention. The procedures and content of SbS are described in more detail in a separate paper on the development of the intervention [13].

Unlike some other SbS trials, participants in the STRENGTHS trials received COD instead of weekly scheduled contacts with e-helpers. Contact was optional via an in-app messaging system, where trained and supervised non-specialist e-helpers—native Syrian Arabic speakers with a background in psychology based in Germany—offered support to participants in both the Germany and Sweden trials. This was the same as the Egypt trial that was conducted partially in parallel [15]. The support covered technical issues, referral information, and questions about the intervention or the study. E-helpers were trained to identify adverse events and could flag and escalate cases needing extra support. Country-specific referral information (e.g., crisis hotlines) was available in-app, and e-helpers followed up with participants to provide tailored referrals. The project was supported by an experienced clinical supervisor and overseen by the STRENGTHS Safety Board. For participants who did not request assistance, the intervention remained unguided.

The SbS content was delivered in a narrative format similar to common messaging apps, with each session following a fictional protagonist who received help from a health professional using SbS. At the start of the intervention, participants selected whether they preferred to be addressed as male or female. Based on this selection, the grammatically correct gendered forms were used in Arabic. Participants could either read these conversations or listen to them in audio form. The gender of the fictional protagonist matched the participant’s selection. For example, if a participant chose female, the story featured a female protagonist with corresponding illustrations and voice recordings. At the end of each session, the fictional health professional character addressed the participant directly, explaining new exercises and instructing them to practice the techniques before the next session unlocked after 3 days. As part of the cultural and contextual adaptation of SbS, participants were presented with a choice between an older or younger protagonist to match their personal circumstances better and with options to customize the protagonist’s appearance [24].

The CAU arm did not receive access to the SbS intervention. Participants in this group were free to use any services available to them in their country of residence (or online), ranging from no care to specialized care. In addition, a short information session presented selected psychoeducative content from the first SbS session in a neutral text-based format (i.e., without using a fictional health professional), omitting the narrative components and illustrations characteristic of the SbS intervention sessions.

### Outcomes

Data assessments were fully integrated into the app through audio-supported questionnaires at baseline, six weeks after baseline (post), and three months after post (follow-up). The primary endpoint for the intention-to-treat (ITT) analyses was the three-month follow-up. Participants received compensation for completing the post- and follow-up questionnaires through vouchers worth 20 euros (Germany) or 200 Swedish kronor (Sweden) per assessment.

Psychological distress and psychosocial functioning were the primary outcomes. Psychological distress was measured using the Arabic version of the 25-item Hopkins Symptom Checklist (HSCL-25) [28] on a 1–4 scale, with higher scores indicating greater distress. The HSCL-25 was validated in Arabic samples [28, 29]. The baseline reliability (Cronbach’s α) of the questionnaire was 0.92 in Germany and 0.93 in Sweden. Psychosocial functioning was assessed using the Arabic version of the 12-item WHO Disability Assessment Schedule (WHODAS 2.0) [30] on a 1–5 scale, where higher scores indicate lower functioning. The WHODAS 2.0 was validated as a screening tool in an Arabic sample [31]. The baseline reliability (Cronbach’s α) of the assessment was 0.84 in Germany and 0.86 in Sweden.

Symptoms of PTSD and the burden of self-defined problems were secondary outcomes. PTSD symptoms were measured using the Arabic version of the 9-item short form of the PTSD Checklist for DSM-5 (PCL-5 short) [32] on a 0–4 scale, with higher scores indicating a higher symptom load. Although the Arabic PCL-5 short has not been validated, the full Arabic PCL-5 has shown psychometric properties comparable to those reported in English-language validation studies [33, 34]. The baseline Cronbach’s α for the PCL-5 short was 0.87 (Germany) and 0.91 (Sweden). The burden of self-defined problems was measured with the Psychological Outcomes Profiles Scale (PSYCHLOPS) [35]. This measure asks participants to identify two current problems in their daily lives and then assesses the impact of each problem on functioning and well-being. Responses are rated on a 0–5 scale, with higher scores indicating a greater perceived burden. The PSYCHLOPS was validated in several studies [36, 37], but not yet in Arabic samples. The baseline Cronbach’s α for the questionnaire was 0.81 (Germany) and 0.86 (Sweden).

Additional measures included sociodemographic questions (gender, age, education level, marital status, and occupational status). In addition, the number of messages sent through the in-app COD feature was passively tracked.

### Analyses

Both studies were designed to detect an effect size of Hedges’ g = 0.4 with power = 0.90 and α = 0.05 (two-sided). The anticipated drop-out rate at the 3-month follow-up was 50%, resulting in a minimum sample size of 532 participants (266 per arm). All analyses were conducted in R version 4.3.3 [38]. The complete ITT datasets were used for the analyses, conducted separately for each trial. To estimate changes in primary and secondary outcomes from baseline to post-intervention and to the 3-month follow-up (the primary endpoint), linear mixed models (LMM) were employed. The models included study condition, time (categorical: baseline, post, and 3-month), and the interaction between study condition and time as fixed effects. Baseline measurements of the outcomes were also included as a fixed effect to estimate within-subject changes. Demographic variables (gender, age, marital status, education, occupation) were added as covariates. Participant ID was specified as a random effect to account for individual variability in outcome trajectories over time. Using the same LMM specifications, exploratory per-protocol analyses were conducted with participants who received the intended SbS dose of at least 4 out of 5 sessions (SbS + CAU arm) or who completed the information session (CAU arm).

For all LMMs, model estimates were aggregated across 100 imputed datasets created using the Multivariate Imputation by Chained Equations (mice) package in R [39]. To improve imputation accuracy, baseline scores and demographic variables were included as predictors. The estimates from each imputed dataset were then pooled using Rubin’s Rules [40]. Effect sizes (Hedges’ g) were calculated using the aggregated estimates from the imputed datasets and interpreted according to convention (0.2 = small effect, 0.5 = moderate effect, 0.8 = large effect) [41]. All statistical tests were two-tailed, with *p* < .05 considered statistically significant.

## Results

### Sample characteristics

**Fig. 1:**
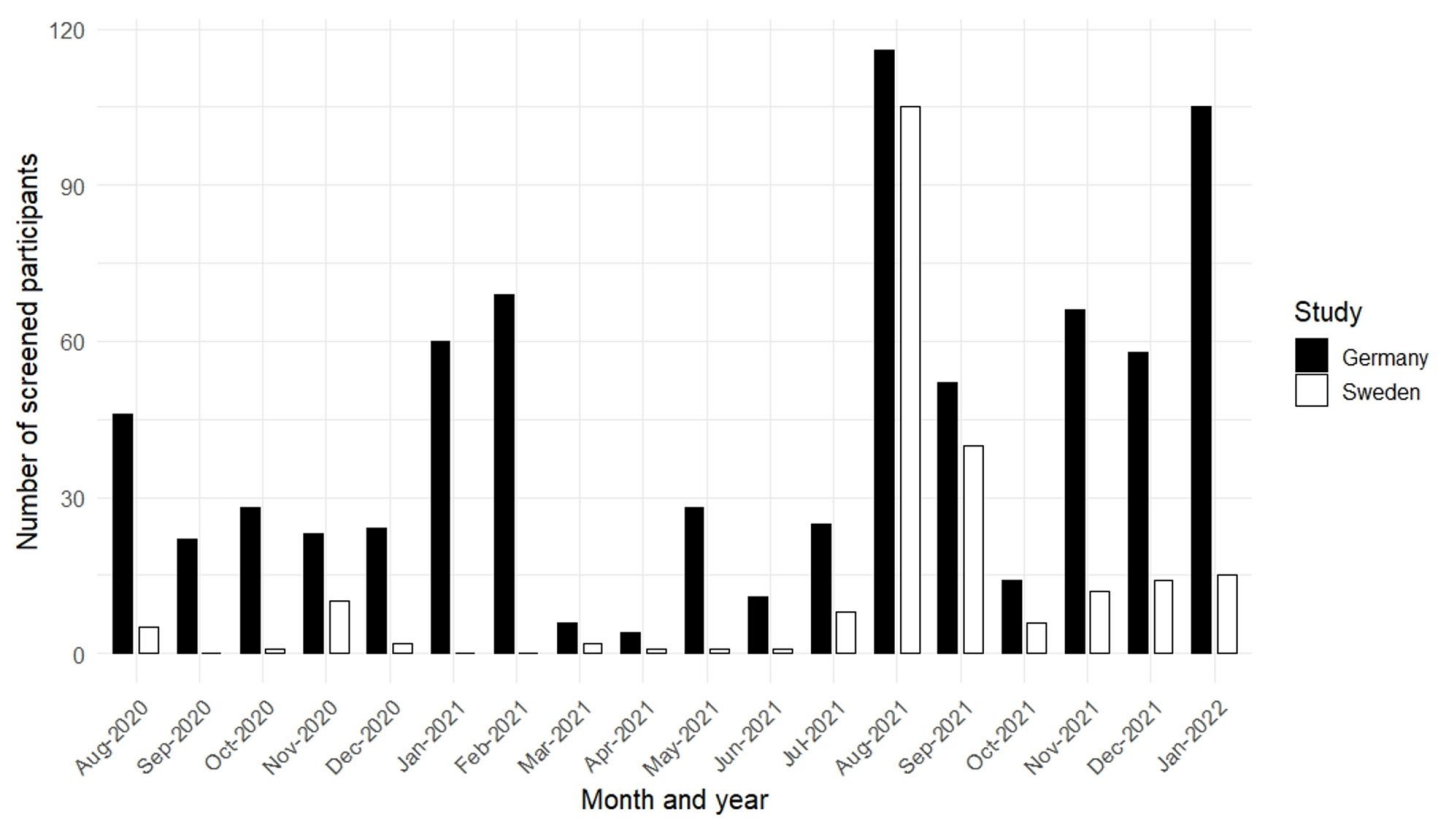
Study recruitment numbers for the Germany and Sweden RCTs

Recruitment in Germany and Sweden took place between August 2020 and January 2022. In Germany, the minimum sample size was reached after screening *N* = 963 potential participants, of whom *N* = 559 were randomized. The slight oversampling was due to delayed baseline completion. In Sweden, the minimum sample size was not reached, and recruitment was stopped with *N* = 184 participants randomized after screening *N* = 262 potential participants because of time constraints. Figure 1 shows the monthly screening numbers. All recruitment occurred during the COVID-19 pandemic. Owing to travel restrictions and reduced face-to-face services for refugees, most outreach relied on social media groups and targeted advertisements aimed at Syrian refugees in Germany and Sweden. This strategy produced a steady inflow in Germany, averaging *n* = 29 participants per month between August 2020 and July 2021, but yielded only about *n* = 3 participants per month in Sweden. In August 2021, a shift to a social-media-influencer-focused campaign substantially increased screening rates, averaging *n* = 69 participants per month in Germany and *n* = 32 per month in Sweden from August 2021 to January 2022. However, after an initial surge, recruitment in Sweden dropped rapidly, ultimately falling short of the target sample size.

Table 1 provides an overview of the demographic characteristics and baseline levels of psychological distress, functioning, PTSD symptoms, and self-defined problems of the participants. The average age of participants in Germany was 30.66 (SD = 8.20), with 69.6% female participants. In Sweden, the average age was 32.85 (SD = 10.00), with 80.4% female participants. In both countries, the majority had started secondary or higher education (Germany: 93.4%, Sweden: 91.8%), were either in paid work (Germany: 25.0%, Sweden: 34.2%) or students (Germany: 30.6%, Sweden: 33.7%). More than half of the participants were married (Germany: 67.3%, Sweden: 80.4%).

**Table 1:** Demographic and baseline characteristics for the Germany and Sweden RCTs

	Germany				Sweden		
	SbS + CAU (*n* = 299)	CAU (*n* = 260)	Total (*n* = 559)		SbS + CAU (*n* = 95)	CAU (*n* = 89)	Total (*n* = 184)
M (SD)							
**Age**	30.61 (8.03)	30.72 (8.40)	30.66 (8.20)		33.39 (9.99)	32.27 (10.03)	32.85 (10.00)
**HSCL-25**	2.54 (0.58)	2.52 (0.57)	2.53 (0.58)		2.45 (0.64)	2.49 (0.63)	2.47 (0.64)
**WHODAS**	32.12 (8.40)	31.86 (8.27)	32.00 (8.33)		30.29 (8.55)	31.66 (9.19)	30.96 (8.87)
**PCL-5**	17.99 (7.33)	18.65 (7.23)	18.30 (7.29)		16.57 (8.01)	17.99 (7.93)	17.26 (7.98)
**PSYCHLOPS**	15.84 (3.84)	16.33 (3.71)	16.07 (3.78)		15.41 (4.24)	15.65 (4.38)	15.53 (4.30)
% (n)							
**Female**	71.9% (215)	66.9% (174)	69.6% (389)		82.1% (78)	78.7% (70)	80.4% (148)
**Marital status**							
Never married	29.4% (88)	33.1% (86)	31.1% (174)		17.9% (17)	23.6% (21)	20.7% (38)
Married	55.9% (167)	50.0% (130)	53.1% (297)		68.4% (65)	62.9% (56)	65.8% (121)
Separated	5.0% (15)	5.4% (14)	5.2% (29)		3.2% (3)	3.4% (3)	3.3% (6)
Divorced	4.7% (14)	8.8% (23)	6.6% (37)		6.3% (6)	6.7% (6)	6.5% (12)
Widowed	0.7% (2)	0.4% (1)	0.5% (3)		0.0% (0)	0.0% (0)	0.0% (0)
Other	4.3% (10)	2.3% (6)	3.4% (19)		4.2% (4)	3.4% (3)	3.8% (7)
**Education** ^**1**^							
No education	1.7% (5)	1.2% (3)	1.4% (8)		1.1% (1)	2.2% (2)	1.6% (3)
Primary	5.4% (16)	3.8% (10)	4.7% (26)		5.3% (5)	7.9% (7)	6.5% (12)
Secondary	40.8% (122)	37.7% (89)	39.4% (220)		35.8% (34)	36.0% (32)	35.9% (66)
University	45.5% (136)	50.0% (130)	47. 6% (266)		50.5% (48)	50.6% (45)	50.5% (93)
Technical	6.7% (20)	6.2% (16)	6.4% (36)		7.4% (7)	3.4% (3)	5.4% (10)
Other	0.0% (0)	1.2% (3)	0.5% (3)		0.0% (0)	0.0% (0)	0.0% (0)
**Occupation**							
Paid work	23.1% (69)	27.3% (71)	25.0% (140)		35.8% (34)	32.6% (29)	34.2% (63)
Self-employed	6.7% (20)	5.0% (13)	5.9% (33)		2.1% (2)	2.2% (2)	2.2% (4)
Unpaid work	0.7% (2)	0.0% (0)	0.4% (2)		0.0% (0)	0.0% (0)	0.0% (0)
Student	31.1% (93)	30.0% (78)	30.6% (171)		31.6% (30)	36.0% (32)	33.7% (62)
Homemaker	18.7% (56)	15.4% (40)	17.2% (96)		10.5% (10)	5.6% (5)	8.2% (15)
Retired	0.3% (1)	0.0% (0)	0.2% (1)		1.1% (1)	1.1% (1)	1.1% (2)
Unemployed	14.7% (44)	18.1% (47)	16.3% (91)		12.6% (12)	13.5% (12)	13.0% (24)
Other	4.3% (13)	4.2% (11)	4.3% (24)		6.3% (6)	9.0% (8)	7.6% (14)

^1^highest education level started

### Study and intervention completion

**Fig. 2:**
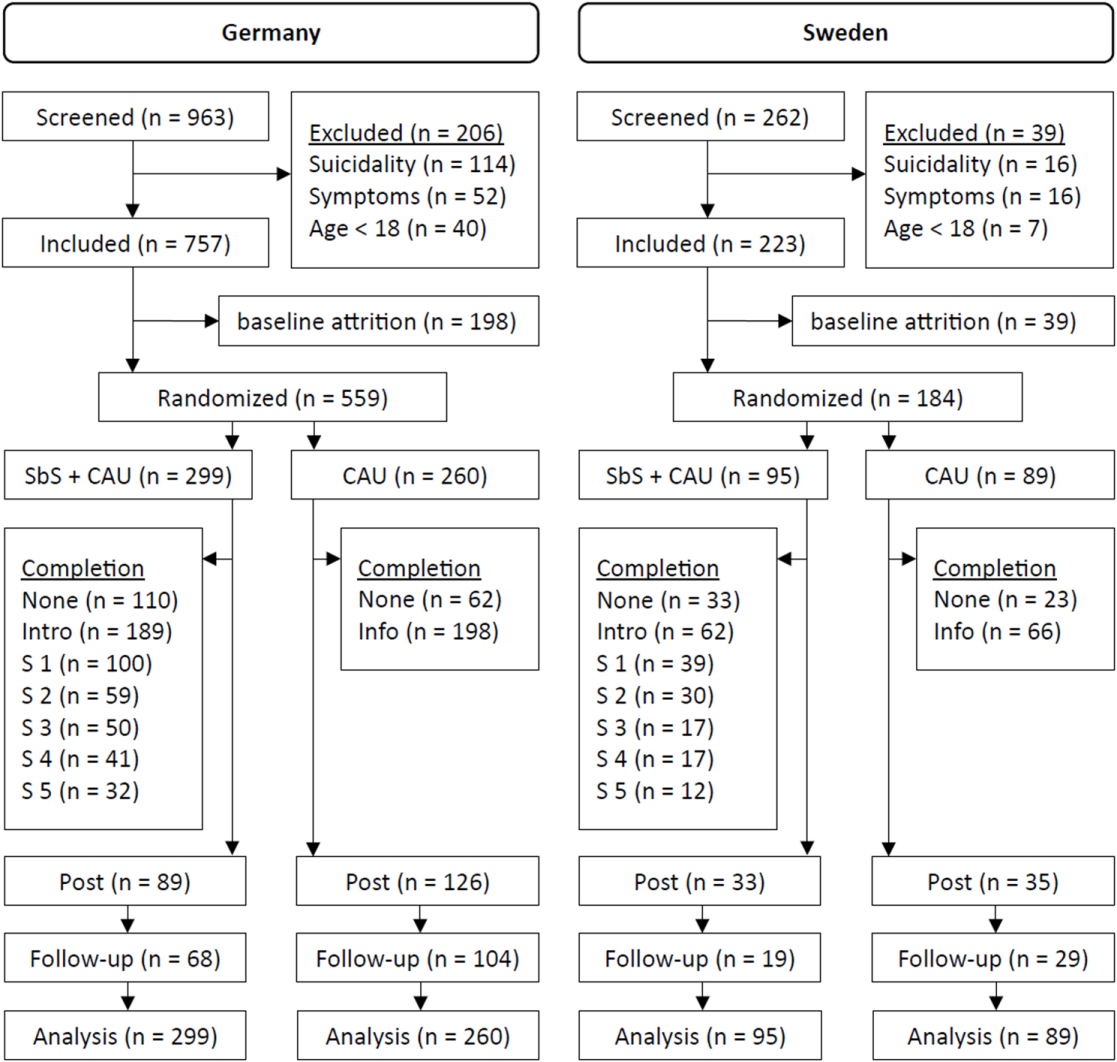
Flowchart for the Germany and Sweden RCTs

Figure 2 provides details on participant flow, session completion, and study drop-out for both trials. In Germany, *n* = 299 (53.5%) participants were randomized to the intervention (SbS + CAU) arm and *n* = 260 (46.5%) to the CAU arm. Study dropout in Germany was higher than expected, with *n* = 215 (38.6%) completing the post-assessment and *n* = 172 (30.7%) completing the follow-up. A similar pattern was found in Sweden where *n* = 95 participants (51.6%) were randomized to the SbS + CAU arm and *n* = 89 (48.4%) to the CAU arm with *n* = 68 (37.0%) completing the post assessment and *n* = 48 (26.1%) completing the follow-up.

The intervention completion rate (4 out of 5 sessions) was 13.7% in Germany and 17.9% in Sweden. In-depth analyses on drop-out rates in the SbS + CAU arm revealed that drop-outs were especially common at the very beginning of the intervention. After the introduction session, the dropout rate in the German trial was 36.8%, increasing to 66.6% after session 1 and 80.3% after session 2, then rising more slowly to 83.3% after session 3, 86.3% after session 4, and 89.3% after session 5. In the Swedish trial, the post-introduction dropout rate was 34.7%, increasing to 58.9% after session 1, 68.4% after session 2, 82.1% after sessions 3 and 4, and 87.4% after session 5. The COD option was used by *n* = 45 participants in Germany (15.1%), reaching out to the e-helper team by sending a message using the app’s messaging system. In Sweden, a total of *n* = 8 participants (8.4%) utilized the COD feature.

### Primary and secondary outcomes

The ITT analyses were pooled across all imputed datasets and are summarized in Tables 2 and 3. In both study arms, psychological distress, functioning, PTSD symptoms, and self-defined problems improved over time. However, no statistically significant differences between the study arms were found on the primary and secondary outcome measures. The observed effect sizes were small, indicating minimal impact of SbS on any of the outcomes in the ITT samples.

**Table 2:** Pooled results from LMMs in the Germany RCT (*N* = 559, based on MI)

Outcomes		Descriptive statistics, Pooled^1^ M (SD)				Linear mixed model analysis^2^, Pooled results^3^		
	Time point	SbS + CAU	*n*	CAU	*n*	Mean diff. (95% CI)	*p*-value	Effect size^4^
Primary								
**HSCL-25** (psych. distress)	Baseline	2.54 (0.58)	299	2.52 (0.57)	260			
	Post	2.41 (0.67)	89	2.50 (0.62)	126	-0.11 (-0.26, -0.05)	.17	0.17
	Follow-up	2.27 (0.70)	68	2.35 (0.67)	104	-0.10 (-0.26, -0.07)	.25	0.15
**WHODAS** (functioning)	Baseline	32.12 (8.40)	299	31.86 (8.27)	260			
	Post	31.17 (9.51)	89	29.78 (8.66)	126	1.13 (-0.96, 3.22)	.29	0.12
	Follow-up	29.64 (9.94)	68	28.42 (9.20)	104	0.97 (-1.35, 3.29)	.41	0.10
Secondary								
**PCL-5 (short)** (PTSD symptoms)	Baseline	17.99 (7.33)	299	18.65 (7.23)	260			
	Post	17.24 (8.15)	89	17.45 (7.87)	126	-0.45 (-1.34, 2.24)	.62	0.06
	Follow-up	15.86 (8.24)	68	16.89 (8.20)	104	-0.37 (-2.43, 1.70)	.72	0.04
**PSYCHLOPS** (self-defined problems)	Baseline	15.84 (3.84)	299	16.33 (3.71)	260			
	Post	12.98 (5.32)	89	13.07 (4.94)	126	-0.40 (-0.93, 1.73)	.55	0.08
	Follow-up	11.29 (5.34)	68	12.04 (5.28)	104	-0.26 (-1.66, 1.14)	.71	0.05

^1^Pooled descriptive statistics across all imputed datasets; ^2^As covariates the models included: baseline score, gender, age, marital status, education, and occupation; ^3^Treatment effects were pooled based on multiple imputations (100), assuming missing at random, using progressive mean matching (PMM); ^4^Hedges’ g effect sizes were derived by combining multiple imputation estimates using Rubin’s rules

**Table 3:** Pooled results from LMMs for the Sweden RCT (*N* = 184, based on MI)

Outcomes		Descriptive statistics, Pooled^1^ M (SD)				Linear mixed model analysis^2^, Pooled results^3^		
	Time point	SbS + CAU	*n*	CAU	*n*	Mean diff. (95% CI)	*p*-value	Effect size^4^
Primary								
**HSCL-25** (psych. distress)	Baseline	2.45 (0.64)	95	2.49 (0.63)	89			
	Post	2.12 (0.70)	33	2.34 (0.65)	35	-0.18 (-0.45, 0.09)	.20	0.26
	Follow-up	2.16 (0.78)	19	2.08 (0.68)	29	0.12 (-0.29, 0.53)	.57	0.16
**WHODAS** (functioning)	Baseline	30.29 (8.55)	95	31.66 (9.19)	89			
	Post	27.40 (10.82)	33	28.83 (9.62)	35	-0.07 (-4.33, 4.20)	.98	0.01
	Follow-up	27.85 (11.64)	19	27.07 (10.15)	29	2.16 (-3.49, 7.81)	.45	0.20
Secondary								
**PCL-5 (short)** (PTSD symptoms)	Baseline	16.57 (8.01)	95	17.99 (7.93)	89			
	Post	14.05 (8.74)	33	14.87 (8.03)	35	-0.60 (-2.65, 3.85)	.72	0.07
	Follow-up	14.85 (10.32)	19	13.45 (9.22)	29	2.82 (-2.21, 7.86)	.27	0.29
**PSYCHLOPS** (self-defined problems)	Baseline	15.41 (4.24)	95	15.65 (4.38)	89			
	Post	11.46 (5.33)	33	13.02 (5.06)	35	-1.32 (-3.53, 0.90)	.24	0.25
	Follow-up	10.54 (5.71)	19	10.39 (5.28)	29	0.39 (-2.53, 3.31)	.79	0.07

^1^Pooled descriptive statistics across all imputed datasets; ^2^As covariates the models included: baseline score, gender, age, marital status, education, and occupation; ^3^Treatment effects were pooled based on multiple imputations (100), assuming missing at random, using progressive mean matching (PMM); ^4^Hedges’ g effect sizes were derived by combining multiple imputation estimates using Rubin’s rules

### Exploratory analyses

The exploratory per protocol analysis combined data from both studies, yielding a total of *n* = 322 completers, of whom *n* = 264 (82.0%) were in the CAU arm, which provided only a short information session instead of the 5 SbS sessions. As summarized in Table 4, among these completers, significantly lower mean scores for psychological distress (HSCL-25) were observed in the SbS + CAU arm at post and at the 3-month follow-up. At the primary endpoint, the mean difference was − 0.22 (95% CI -0.41, -0.02), corresponding to an effect size of d = 0.31. A similar effect was found for PTSD symptoms at the 3-month follow-up, with a mean difference of -2.47 (95% CI -4.89, -0.05) and an effect size of d = 0.27. No additional statistically significant differences were found.

**Table 4:** Pooled results from LMMs for the per-protocol analyses1 in the Germany and Sweden RCTs combined (*N* = 322, based on MI)

Outcomes		Descriptive statistics, Pooled^2^ M (SD)				Linear mixed model analysis^3^, Pooled results^4^		
	Time point	SbS + CAU	*n*	CAU	*n*	Mean diff. (95% CI)	*p*-value	Effect size^5^
Primary								
**HSCL-25** (psych. distress)	Baseline	2.54 (0.64)	58	2.50 (0.60)	264			
	Post	2.11 (0.81)	55	2.43 (0.64)	151	-0.38 (-0.59, -0.17)	< .001	0.55
	Follow-up	2.02 (0.79)	48	2.18 (0.68)	122	-0.22 (-0.41, -0.02)	< .05	0.31
**WHODAS** (functioning)	Baseline	31.64 (8.93)	58	31.67 (8.51)	264			
	Post	27.42 (11.70)	55	29.22 (8.71)	151	-1.85 (-4.79, 1.09)	.22	0.19
	Follow-up	26.38 (11.28)	48	27.28 (9.84)	122	-0.89 (-3.51, 1.72)	.50	0.09
Secondary								
**PCL-5 (short)** (PTSD symptoms)	Baseline	17.67 (8.24)	58	18.38 (7.48)	264			
	Post	13.97 (9.72)	55	16.68 (7.85)	151	-2.14 (-4.82, 0.55)	.12	0.25
	Follow-up	14.10 (10.25)	48	17.20 (8.46)	122	-2.47 (-4.89, -0.05)	< .05	0.27
**PSYCHLOPS** (self-defined problems)	Baseline	15.31 (4.48)	58	16.09 (4.03)	264			
	Post	11.57 (6.03)	55	13.05 (4.91)	151	-0.72 (-2.44, 1.00)	.41	0.14
	Follow-up	9.47 (5.81)	48	10.91 (5.31)	122	-0.62 (-2.24, 1.00)	.45	0.11

^1^Per protocol completers were defined as having completed Session 4 (SbS + CAU arm) or the information session (CAU arm); ^2^Pooled descriptive statistics across all imputed datasets; ^3^As covariates the models included: baseline score, gender, age, marital status, education and occupation; ^4^Treatment effects were pooled based on multiple imputations (100), assuming missing at random, using progressive mean matching (PMM); ^5^Hedges’ g effect sizes were derived by combining multiple imputation estimates using Rubin’s rules

## Discussion

This paper presented the results of two parallel trials evaluating a potentially scalable self-guided version of WHO’s DMHI SbS for Syrian refugees in Germany and Sweden—representing the first time SbS has been tested in high-income countries. The trials were conducted alongside other trials with a self-guided version of SbS for Syrian refugees in Egypt [15] and a guided version of SbS in Lebanon [25, 26], providing a unique opportunity to compare different implementation formats across diverse settings.

The key finding of our trials among Syrian refugees in Germany and Sweden is that no statistically significant time x condition effects were detected in the ITT analyses for psychological distress, functioning, PTSD symptoms, or self-defined problems. This contrasts with prior SbS trials that found medium effects on similar outcomes among Syrians and Lebanese in Lebanon when offered with weekly 15 min of guidance and small effects for Syrian refugees in Egypt when offered self-guided with COD [15, 25, 26].

Both trials had very high intervention dropout rates—86.3% in Germany and 82.1% in Sweden—leading to a low intervention dose for most participants. These dropout rates exceeded those seen in earlier SbS studies in Lebanon (60–70%). Such low retention is not unusual in low-threshold, smartphone-delivered interventions [42], especially when they are unguided and rely on online, anonymous recruitment [43]. Exploratory per protocol analyses pointed to a trend of greater improvements in psychological distress and PTSD symptoms among participants who completed 4 or 5 (out of 5) SbS sessions. While these findings must be interpreted cautiously given the highly selective sub-sample, they suggest that improving adherence—potentially by incorporating more guidance—may be necessary to achieve outcomes similar to those found in other SbS trials in Lebanon and Macau [44] or trials on digital interventions for migrants in Sweden [45, 46] that all included weekly scheduled support.

As also observed in the Egypt trial with 9.4% COD usage [15], the COD guidance model was rarely utilized in Germany (15.1%) and Sweden (8.4%), leaving the intervention fully unguided for most participants. It appears that a weekly-guidance version in a lower-middle-income country (Lebanon) achieved the strongest results, followed by a self-guided version with low COD usage in another lower-middle-income country (Egypt). By comparison, using the same guidance model as in Egypt but in high-income settings (Germany and Sweden) yielded the smallest effects. For DMHI some form of human support and contact may facilitate engagement [16] and a recent trial amongst migrants in Italy supports this notion [47]. It showed another WHO digital self-help intervention was effective, as part of a stepped care model, when offered with the same guidance model used in the SbS Lebanon trials. This suggests that for some populations, such as refugees, guidance may be critical for increasing engagement.

It is important to note differences relative to previous SbS trials. For instance, in Egypt only 12.6% of participants had begun university studies, compared to 47.6% in Germany and 50.5% in Sweden. Unemployment rates were also much lower in Germany (16.3%) and Sweden (13.0%) than in Lebanon (58.7%) [25]. Coupled with broader mental healthcare options in Germany and Sweden, these sociodemographic distinctions may influence how a digital intervention like SbS is perceived across different contexts. Additionally, participants in Lebanon were screened for moderate or severe depressive symptoms (PHQ-9 ≥ 10), whereas the STRENGTHS trials screened for elevated psychological distress (K10 > 15). Because SbS primarily focuses on depressive symptoms, enrolling participants with higher levels of depression may partly explain the stronger effects observed in Lebanon.

Across all SbS trials, a higher proportion of participants were female, which is unusual in digital interventions for refugees where men are often overrepresented [18]. This pattern was seen among Syrians in Lebanon (58.3% female) [25], Syrians in Egypt (67.3% female) [15], the Lebanese population (69.9% female) [26], as well as the trials in Germany (69.4%) and Sweden (80.4%). However, this gender distribution aligns with digital mental health trials in other populations worldwide, where samples were found to include 60% to 86.4% female participants [48], a pattern also observed in psychotherapy trials more broadly [49]. This is commonly attributed to higher mental health literacy and lower stigma among women [48]. Another possible reason is that the recruitment approaches worked better for women. Social media was a major recruitment channel and may have introduced bias. For example, collaborating with lifestyle influencers may have reached more female audiences. However, in the Egypt and Lebanon trials, other recruitment strategies were used. This factor may explain the exceptionally high percentage of female participants in the Swedish trial, where most of the sample was recruited through influencer-driven social media communities. In terms of adherence, it has been noted that low intervention usage should not always be equated with low adherence to self-help strategies, especially in interventions centered on mental health recovery narratives [50], which are a key feature of SbS. Participants may have deliberately avoided reading more about the SbS protagonist’s challenges in later sessions in order to focus on their own problems or practice learned techniques outside the app. Low usage also raises questions about the relevance of the SbS content in high-income countries. This highlights the challenges of creating highly scalable, globally deployable interventions, that lack robust contextual as well as cultural adaptation. Since much of SbS’s cultural tailoring was developed for Lebanon [24], the intervention narratives had the strongest contextual alignment in the Lebanon trials, followed by Egypt, where the socioeconomic environment may have more closely resembled that of Lebanon than that for Syrian refugees in Germany or Sweden, and this may have limited engagement. Further adapting SbS content to typical postmigration difficulties and psychosocial needs in Western Europe may be necessary to improve its relevance, for instance by drawing on experiences and challenges from the cultural adaptation of SbS for Albanian-speaking immigrants in Switzerland and Germany [51]. It is noteworthy that dropout rates were highest at the start, particularly during and after the introduction session. This session provided a basic overview of the intervention, introduced the protagonists, and included a brief audio exercise. Potential reasons in both trials align with other SbS studies: (1) low perceived relevance during and after completing the introduction session, (2) the high number of baseline questionnaires, (3) general lack of time to continue with the intervention, (4) difficulties navigating the app, and (5) technical issues [15, 52]. In an anonymous, unguided setup, participants could easily sample the program and faced no barriers to discontinuing use, which is an advantage for reach but may be a disadvantage for adherence. Approaches emphasizing psychosocial support—such as the Swiss Red Cross Sui app [53]—may offer promising examples by addressing both everyday difficulties and mental health concerns, which can be further explored in trials that also investigate the role of guidance. One such approach involves closely integrating digital and in-person support within blended-care settings for refugees, which may help increase adherence and address treatment gaps—particularly where language barriers exist [54].

Beyond socioeconomic factors, implementation in Germany and Sweden also differed regarding its integration within existing refugee support structures. Recruitment proved difficult, especially in Sweden. COVID-19–related restrictions limited face-to-face outreach, making it harder to build trust with possible participants without a partner organization (which was different in the Egypt trial). Traditional social media advertising was only modestly effective; while an influencer-based campaign substantially raised screening numbers, this effect was confined to the campaign period and did not produce lasting recruitment gains. However, considering the absolute number of Syrian refugees and asylum seekers in Sweden (116,743) and Germany (675,490) in 2021 [55], the approximate proportion of the target population screened for the trials was slightly higher in Sweden (0.22%) than in Germany (0.14%). This suggests that Sweden’s recruitment challenges may have stemmed from the lower absolute number of refugees rather than a reduced overall reach. Unlike parallel trials in Lebanon and Egypt, which partnered with public authorities and non-governmental organizations, SbS in Germany and Sweden lacked continuous embedding in trusted infrastructures, underscoring the value of ongoing partnerships to maintain awareness and consistent recruitment.

### Strengths and limitations

The study is limited by high intervention and assessment dropout rates that constrain the interpretability of results. In Sweden, the target sample size was not reached, affecting the statistical power of the analyses. The representativeness of the findings is limited by the overrepresentation of female participants, particularly in the Swedish sample. The COVID-19 pandemic further complicated procedures, which should be kept in mind when interpreting the findings. COVID-19 measures varied substantially between Germany and Sweden [56, 57], but research indicates a general mental health decline in most countries, that then improved over time [58], potentially obscuring intervention-related effects. A further limitation was the fact that one e-helper team based in Germany responded to participants in both countries. This was done due to the high resource costs of training and maintaining teams providing guidance. However, because the overall utilization of our contact-on-demand offer was very low, we did not reach a point at which additional teams would have been justified in terms of e-helper capacity. Strengths of these studies include their real-world conditions and—specifically for the Germany trial—a large sample size. They also form part of a unique effort to evaluate SbS with different guidance formats across multiple countries for the same target population. The availability of multiple studies enables more robust comparisons and offers clearer insight into the question, “How low can we go?” regarding guidance in SbS. However, additional head-to-head trials of guided versus unguided versions of SbS in different populations may provide clearer insights into this issue.

## Conclusions

Digital interventions must navigate challenges related to engagement, adherence, and contextual relevance. The SbS trials in Germany and Sweden show that attempting to increase scale by reducing human input and contextual adaptation too much may jeopardize their intensity and intended clinical impact among refugees in high-income settings. Overall, the findings suggest that SbS worked best for refugees when implemented with weekly guidance in Lebanon but still worked to some degree in a self-guided format in Egypt. However, the current self-guided version of SbS may be insufficient for refugees in high-income countries like Germany and Sweden, where dropout and effect sizes indicate that additional adaptations or greater guidance may be necessary to match local needs and circumstances. Moving forward, deeper process evaluations of SbS implementation, including qualitative assessments of user expectations and feedback, may be key to understanding where unguided versions of the intervention are best suited. Despite these needed refinements, the successful parallel implementation of three RCTs as part of the STRENGTHS project underscores SbS’s scalability.

## Data Availability

The data collected for this study involves sensitive information obtained from Syrian refugees. Due to ethical concerns regarding the potential misuse of this data by individuals or groups with political agendas, we are unable to make it publicly available. The potential for this data to be misinterpreted or used out of context poses significant risks, including the possibility of further marginalization or stigmatization of refugee populations. In agreement with the European Commission, all members of the STRENGTHS consortium have agreed that access to the data collected under the EU-funded STRENGTHS project is instead ensured via a central repository managed by the Vrije Universiteit Amsterdam (VU). Access to this data is available upon reasonable request to the STRENGTHS consortium. Please note that access may be restricted for third parties if it conflicts with data protection laws applicable in the participating countries or with relevant EU legislation. Interested researchers can contact data steward Alex van der Jagt at apc.vander.jagt@vu.nl for inquiries and to initiate the process.

## Declarations

### STRENGTHS consortium

The STRENGTHS consortium members in alphabetical order are: Ceren Acarturk^10^, Aemal Akhtar^11^, Ibrahim Akinçi^12^, Mhd Salem Alkneme^1,3^, Ammar Alsaod^1^, Ahmed Bawaneh^13^, Martha Bird^14^, Felicity L. Brown^15,16^, Richard Bryant^11^, Sebastian Burchert^1,2^, Pim Cuijpers^5,6^, Anne M. de Graaff^4,5^, Annelieke Drogendijk^17^, Daniela Fuhr^18^, Jonas Maria Hessling^1^, Zeynep Ilkkursun^10^, Mark J.D. Jordans^15,16^, Christine Knaevelsrud^1,2^, Gülşah Kurt^10^, David McDaid^19^, Saara Martinmäki^17^, Cansu Mirzanlı^12^, Trudy Mooren^17^, Naser Morina^20^, A-La Park^19^, Monique Pfaltz^21^, Bayard Roberts^18^, Matthis Schick^20^, Ulrich Schnyder^20^, Marit Sijbrandij^5^, Egbert Sondorp^22^, Julia Spaaij^22^, Frederik Steen^15^, Karine Taha^23^, Peter Ventevogel^24^, Claire Whitney^13^, Nana Wiedemann^14^, Aniek Woodward^22^

^1^ Department of Education and Psychology, Division of Clinical Psychological Intervention, Freie Universität Berlin, Berlin, Germany

^2^ German Center for Mental Health (DZPG), partner site Berlin-Potsdam, Berlin, Germany

^3^ Department of Systemic Psychotherapy, Berlin School of Psychology, Berlin, Germany

^5^ Department of Clinical, Neuro and Developmental Psychology, WHO Collaborating Center for Research and Dissemination of Psychological Interventions, Amsterdam Public Health Research Institute, Vrije Universiteit Amsterdam, Amsterdam, The Netherlands

^6^ Babeș-Bolyai University, International Institute for Psychotherapy, Cluj-Napoca, Romania

^10^Koc University, Istanbul, Turkey

^11^School of Psychology, University of New South Wales, Sydney, NSW, Australia

^12^Refugees and Asylum Seekers Assistance and Solidarity Association, Istanbul, Turkey

^13^International Medical Corps, Amman, Jordan

^14^International Federation of Red Cross and Red Crescent Societies, Copenhagen, Denmark

^15^Research and Development Department, War Child Holland, Amsterdam, The Netherlands

^16^Amsterdam Institute of Social Science Research, University of Amsterdam, Amsterdam, the Netherlands

^17^ARQ International, Amsterdam, The Netherlands

^18^London School of Hygiene and Tropical Medicine, London, UK

^19^London School of Economics and Political Science, London, UK

^20^University of Zurich, Zurich, Switzerland

^21^Mid Sweden University, Sundsvall, Sweden

^22^KIT Royal Tropical Institute, Amsterdam, The Netherlands

^23^War Child Lebanon, Beirut, Lebanon

^24^United Nations High Commissioner for Refugees, Geneva, Switzerland

## Abbreviations

CAU: Care-as-usual
COD: Contact-on-demand
CONSORT: Consolidated Standards of Reporting Trials
DMHI: Digital Mental Health Interventions
ITT: Intention-to-treat
K10: Kessler Psychological Distress Scale
LMM: Linear Mixed Model
MI: Multiple imputation
PCL-5: Posttraumatic Stress Disorder Checklist for DSM-5
PSYCHLOPS: Psychological Outcomes Profiles Scale
PTSD: Posttraumatic Stress Disorder
SbS: Step-by-Step
WHO: World Health Organization
WHODAS: WHO Disability Assessment Schedule
